# Uric acid regulates NLRP3/IL-1β signaling pathway and further induces vascular endothelial cells injury in early CKD through ROS activation and K^+^ efflux

**DOI:** 10.1186/s12882-019-1506-8

**Published:** 2019-08-14

**Authors:** Wei Yin, Qiao-Ling Zhou, Sha-Xi OuYang, Ying Chen, Yu-Ting Gong, Yu-Mei Liang

**Affiliations:** 1 Department of Nephrology, Hunan Provincial People’s Hospital, The First Affiliated Hospital of Hunan Normal University, No.61, West Jiefang Road, Changsha, 410005 Hunan Province People’s Republic of China; 2 Department of Nephrology, Xiangya Hospital, Central South University, Changsha, 410008 People’s Republic of China

**Keywords:** Uric acid, NLRP3/IL-1β, Early CKD, ROS activation, K^+^ efflux

## Abstract

**Background:**

Chronic kidney disease (CKD) has been considered as a major health problem in the world. Increasing uric acid (UA) could induce vascular endothelial injury, which is closely related to microinflammation, oxidative stress, and disorders of lipids metabolism. However, the specific mechanism that UA induces vascular endothelial cells injury in early CKD remains unknown.

**Methods:**

Human umbilical vein endothelial cells (HUVECs) were cultured and subjected to different concentrations of UA for different periods. Early CKD rat model with elevated serum UA was established. Western blotting and quantitative real-time PCR (qPCR) were applied for measuring protein and mRNA expression of different cytokines. The animals were sacrificed and blood samples were collected for measurement of creatinine, UA, IL-1β, TNF-α, and ICAM-1. Renal tissues were pathologically examined by periodic acid-Schiff (PAS) or hematoxylin-eosin (HE) staining.

**Results:**

The expression of IL-1β, ICAM-1, NLRP3 complexes, and activation of NLRP3 inflammasome could be induced by UA, but the changes induced by UA were partially reversed by siRNA NLRP3 or caspase 1 inhibitor. Furthermore, we identified that UA regulated the activation of NLRP3 inflammasome by activating ROS and K^+^ efflux. In vivo results showed that UA caused the vascular endothelial injury by activating NLRP3/IL-1β pathway. While allopurinol could reduce UA level and may have protective effects on cardiovascular system.

**Conclusions:**

UA could regulate NLRP3/IL-1β signaling pathway through ROS activation and K^+^ efflux and further induce vascular endothelial cells injury in early stages of CKD.

## Background

In recent years, chronic kidney disease (CKD) has become one of the most recognized public health problems worldwide [1, 2]. Hyperuricemia is prevalent in early CKD patients for the reason that the function of kidney excreting UA has been impaired, and UA increases continuously as the further deterioration of renal function [3, 4]. Meanwhile, several studies have demonstrated that hyperuricemia is an independent risk factor of cardiovascular diseases [5–7]. It was reported that CKD is highly consistent with cardiovascular diseases, and the risk of cardiovascular diseases increase as the decline of renal function [8]. In addition, cardiovascular diseases have become the most common complication of CKD and the leading cause of death in CKD patients [9]. Endothelial dysfunction is considered to be one of the major causes of cardiovascular diseases [10]. The increase of UA could significantly damage cardiovascular endothelial cells, and endothelial dysfunction is positively correlated with serum UA level [11]. Moreover, UA may damage vascular endothelial cells via oxidative stress, inflammation and abnormal lipid metabolism [11, 12]. However, the specific mechanism remains unclear.

The NLR family, pyrin domain-containing 3 (NLRP3) inflammasome is a multiprotein complex comprising NLRP3 receptor protein, apoptosis-associated speck-like protein (ASC) and protease caspase 1 [13]. The increase of multiple fractions of NLRP3 has been identified in several diseases such as acute sterile inflammation, CKD and atherosclerosis [13]. UA is reported to be an important signal of NLRP3 inflammasome activation [14]. And activation of NLRP3 inflammasome in vascular endothelial cells eventually leads to endothelial dysfunction and contributes to the development of cardiovascular diseases [13]. Interleukin-1 beta (IL-1β), a member of interleukin-1 (IL-1) cytokine superfamily, can be activated by caspase 1, so activation of NLRP3 inflammasome is very important for the maturation and secretion of IL-1β. Meanwhile, the activation of reactive oxygen species (ROS) and potassium ions (K^+^) efflux also participate in the activation of NLRP3 inflammasome [15]. Therefore, NLRP3/IL-1β pathway may be closely related to vascular endothelial cells injury. However, in early-stage CKD patients whether UA activates NLRP3/IL-1β and further induces vascular endothelial cells injury through ROS activation and K^+^ efflux are still not well known.

Collectively, human umbilical vein endothelial cells (HUVECs) were used to investigate the mechanisms that UA activates NLRP3/IL-1β and further induces vascular endothelial cells injury through ROS activation and K^+^ efflux in vitro, and further validated them in vivo. The aim of this study is to provide a new perspective for clinical drug therapy.

## Methods

### Cells culture and treatment

HUVECs were isolated by collagenase A perfusion from umbilical cords (obtained from the Department of Obstetrics, Hunan Provincial People’s Hospital) and cultured as described [16]. The cells were incubated in 1640 medium at 37 °C with 5% CO_2_. When the adhering cells reached confluence, passage by trypsin digestion was conducted. After 3 to 5 passages, cells were treated by different concentrations of UA (0, 5, 10, 20 mg/dL) for 24 h, then cells were collected for western blotting and quantitative real-time PCR (qPCR). A suitable concentration of UA was chosen to detect the influence of different incubation time (0, 6, 12, 24 h, respectively) on the expression of IL-1β and ICAM-1 by western blotting and qPCR.

### Western blotting

Total proteins in cells or cells culture supernatant were prepared and quantified. Equal contents of protein were loaded on an SDS-PAGE and then transferred electrophoretically to PVDF membranes (Millipore, USA). After blocking with TBST (5% milk), the membranes were incubated with primary antibody obtained from Abcam (1:1000, Hong Kong, China) at 4 °C overnight. After washing, the membranes were incubated with secondary antibody (1:2000) in TBST for 1 h at room temperature. ECL Plus detection system (Millipore, USA) was used for immunodetection, and the density of the bands was detected by Image J software.

### RNA isolation and qPCR

Total RNA was extracted through TRIzol reagent (Invitrogen Life Technologies, USA) and then was reverse-transcribed into cDNA using the Primer Script RT reagent kit (Takara Bio, China). qPCR was performed with SYBR Premix Ex Taq™ II kit (Takara Bio, China). The primers used for IL-1β, caspase 1, NLRP3, and ASC were listed in Table 1. GAPDH was used as a control. The mRNA levels were calculated relative to internal control using 2^-ΔΔCt^ method.

**Table 1:** qPCR primer sequences used in this study (h: human, r: rat)

Gene	Forward primer	Reverse primer
hIL-1β	5’-CTGAGCTCGCCAGTGAAATG-3’	5’-TGTCCATGGCCACAACAACT-3’
rIL-1β	5’-CAGCAGCATCTCGACAAGAG-3’	5’-CATCATCCCACGAGTCACAG-3’
hNLRP3	5’-AAGGCCGACACCTTGATATG-3’	5’-CCGAATGTTACAGCCAGGAT-3’
rNLRP3	5’-GTAGGTGTGGAAGCAGGACT-3’	5’-CTTGCTGACTGAGGACCTGA-3’
hcaspase 1	5’-CTCAGGCTCAGAAGGGAATG-3’	5’-CGCTGTACCCCAGATTTTGT-3’
rcaspase 1	5’-CCGTGGAGAGAAACAAGGAG-3’	5’-GGACAGGATGTCTCCAGGAC-3’
rASC	5’-TGGCTACTGCAACCAGTGTC-3’	5’-GGCTGGAGCAAAGCTAAAGA-3’
hGAPDH	5’-CCAGGTGGTCTCCTCTGA-3’	5’-GCTGTAGCCAAATCGTTGT-3’
rGAPDH	5’-GCAAGTTCAACGGCACAG-3’	5’-GCCAGTAGACTCCACGACAT-3’

### Establishment of early-stage CKD animal model

The animal model was established as described [17]. Briefly, 20 male Sprague-Dawley rats (220~250 g, 6~8 weeks old) were obtained from Hunan Slac Jingda Experimental Animal Co. Ltd. (Changsha, China) and kept in the animal experimental center of the First Affiliated Hospital of Hunan Normal University (Changsha, China). The rats were raised in standard cages, water and food were freely available. After different treatments, animals were anesthetized with an intraperitoneal injection of 2% sodium pentobarbital (45 mg/kg). The region for surgery was shaved and then cleaned with 75% alcohol. The right kidney was exposed through a longitudinal incision under the right costal arch. The renal pedicle was clamped and ligated after separation of renal capsule and perirenal fat. The right kidney was resected with scissors. Tissues and skins were sutured, respectively. Chlortetracycline was then applied to the incision. The entire procedure mentioned above was conducted in Group A (sham operation group, *n* = 5), but nephrectomy was not applied. Animals in Group C (hyperuricemia group, *n* = 5) and D (allopurinol and hyperuricemia group, *n* = 5) were fed with potassium oxonate (OXO) or/and allopurinol. Animals in Group A and B (operation group, *n* = 5) were gavaged with drinking water with the same amount as in Group C and D.

### Kidney histopathological examination

After 10 weeks of gavage treatment, animals were euthanized with prolonged exposure to isofluorane inhalation (Respirator mode: frequency 60–70 bpm, breathing ratio 1:1, tidal volume 2–3 mL/100 g; The induction concentration of isoflurane was 4% and the maintenance concentration was 2%.) and the renal tissues were collected. Renal tissues were fixed by 4% paraformaldehyde for 48 h, embedded in paraffin, and cut into 4 μm thick sections. After deparaffinization and rehydration, the sections were stained separately with periodic acid-Schiff (PAS) staining and hematoxylin-eosin (HE) staining to observe the pathological changes in tubules, glomeruli, and enal interstitial by microscope at 200× magnification.

### Detection of serum creatinine and UA

After 10 weeks of gavage treatment, animals were sacrificed and the blood samples were collected. After centrifugation, the blood samples were used for measurement of UA and creatinine. Measurement of UA and creatinine in serum was performed using an automated clinical chemistry analyzer (TBA-C16000, Toshiba, Japan).

### Influence of UA on the production of ROS

HUVECs were treated by either UA (20 mg/dL) or normal saline for 24 h. Then cells were loaded with 5 μM working solution at 37 °C for 10 min and washed twice with warm buffer according to the experimental protocol of ROS assay kit (Invitrogen). After another 10 min incubation, cells were analyzed with a fluorescence microscope (PerkinElmer, US).

### Measurements of IL-1β, TNF-α, and ICAM-1 by ELISA

The blood samples were obtained as described above, and the measurements of IL-1β, intercellular adhesion molecule-1 (ICAM-1), and tumor necrosis factor-α (TNF-α) were conducted through ELISA kits (eBioscience, San Diego, CA, USA).

### Statistical analysis

Data were shown as the mean ± SD, and analyzed using GraphPad Prism 6.0 (GraphPad Software Inc., San Diego, CA). The unpaired two-tailed *t*-test for comparison between two groups or one-way analysis of variance (ANOVA) followed by Tukey post hoc test for multiple comparison was performed for differences analysis. *P*-value < 0.05 was viewed as statistically significant difference.

## Results

### UA induced the expression of IL-1β and ICAM-1 in HUVECs

**Fig. 1:**
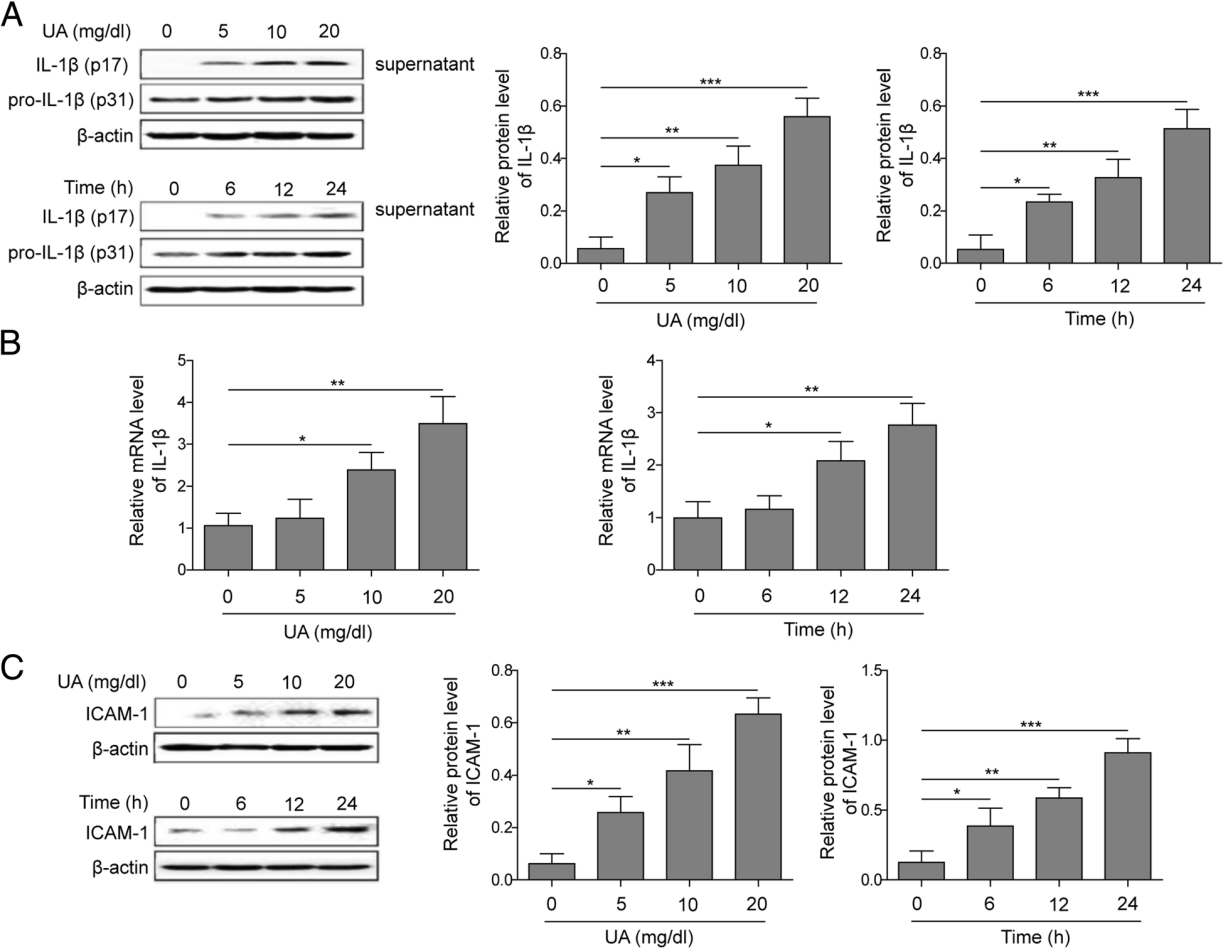
UA induced the expression of IL-1β and ICAM-1 in HUVECs. The cells were treated by UA (5, 10, 20 mg/dL, respectively) for 24 h or UA (6, 12, 24 h, respectively) at 20 mg/dL. **a** Detection of protein expression of IL-1β in cells supernatant by western blotting; **b** Measurement of mRNA expression of IL-1β in cells by qPCR; **c** Measurement of protein expression of ICAM-1 in cells by western blotting. *P < 0.05; **p < 0.01; ***p < 0.001

After treatment with different concentrations of UA or different incubation time, the expression of ICAM-1 and IL-1β in HUVECs was detected by western blotting and qPCR (Fig. 1). UA significantly increased the protein expression of IL-1β in cells culture supernatant and the mRNA level of IL-1β in cells, and the influence presented by concentration and time dependent manner (Fig. 1a, b). However, the expression of IL-1β precursor (pro-IL-1β) showed no obvious change. We also found that with the increase of UA concentration and incubation time, the level of ICAM-1 protein increased consequently (Fig. 1c). Therefore, UA could induce the expression of ICAM-1 and IL-1β in HUVECs.

### UA induced the expression of NLRP3 complexes and activation of NLRP3 inflammasome in HUVECs

**Fig. 2:**
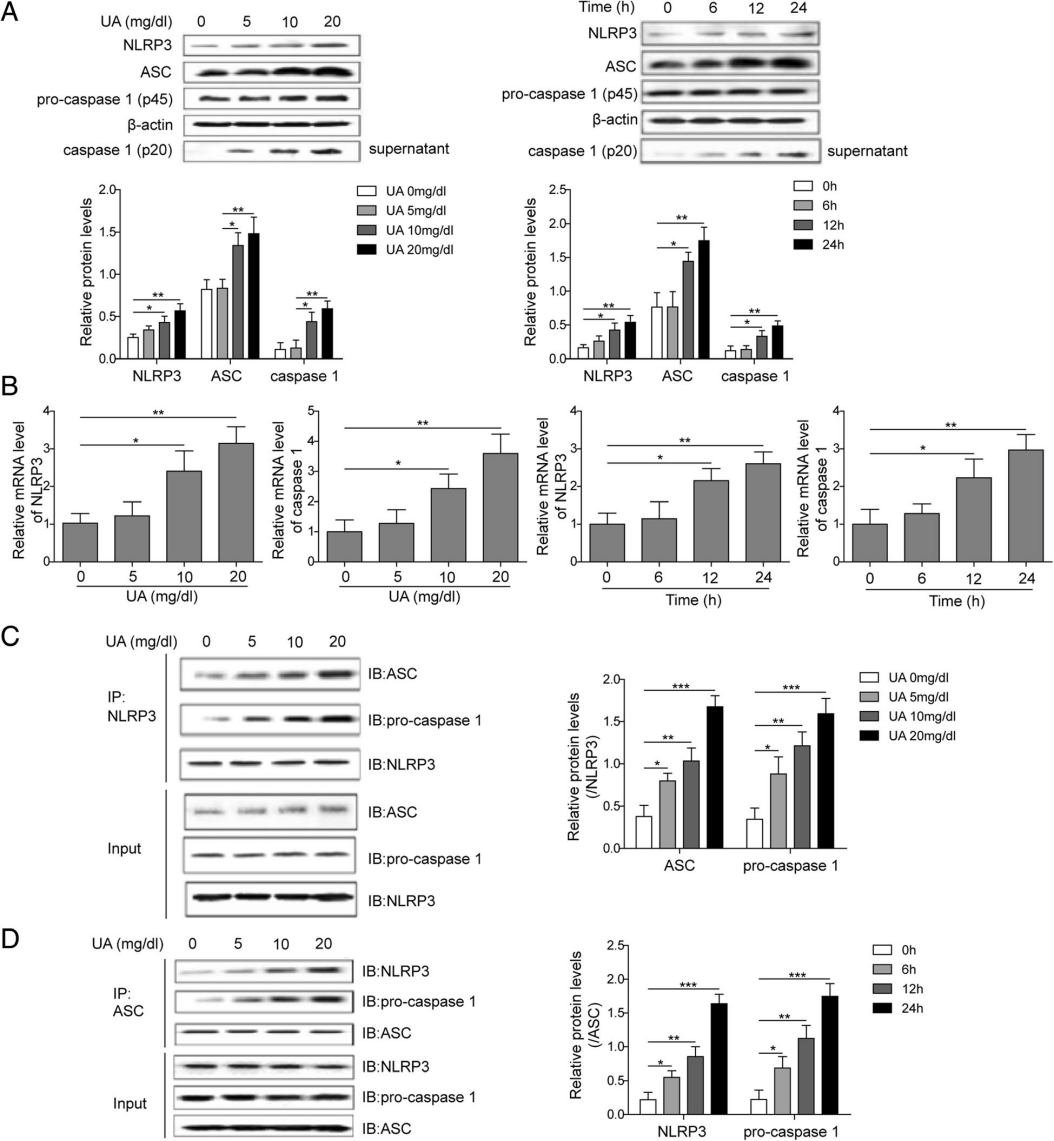
UA induced the expression of NLRP3 complex and activation of NLRP3 inflammasome in HUVECs. The cells were treated by UA (5, 10, 20 mg/dL, respectively) for 24 h or UA (6, 12, 24 h, respectively) at 20 mg/dL. **a** Measurement of the expression of ASC and NLRP3 in cells and the expression of caspase 1 in cells supernatant by western blotting; **b** Measurement of the mRNA expression of caspase 1 and NLRP3 in cells by qPCR; **c** The protein expression of ASC, NLRP3, and pro-caspase 1 in cells lysates extracted by A/G immunomagnetic beaded with NLRP3 antibody was measured by western blotting; **d** The protein expression of ASC, NLRP3, and pro-caspase 1 in cells lysates extracted by A/G immunomagnetic beaded with ASC antibody was measured by western blotting. *P < 0.05; **p < 0.01; ***p < 0.001

In order to investigate the effects of UA on NLRP3 inflammasome, the expression of NLRP3, ASC, and pro-caspase 1 was measured by western blotting and qPCR after the treatment by different concentrations or different incubation time of UA (Fig. 2). We found that high concentrations (10 and 20 mg/dL) of UA or longer incubation time (12 and 24 h) of UA at 20 mg/dL significantly increased the expression of ASC and NLRP3 in cells and the protein level of caspase 1 in medium supernatant. The influence presented concentration and time dependence (Fig. 2a). Accordingly, UA significantly promoted the mRNA expression of NLRP3 and caspase 1 in HUVECs with the concentration and time dependent manner (Fig. 2b).

Furthermore, immunoprecipitation and Western blotting were used to detect the effects of UA treatment on the binding of components in NLRP3 inflammasome. The cells lysates were extracted by A/G immunomagnetic beaded with NLRP3 antibody, then the protein expression of ASC, NLRP3, and pro-caspase 1 was measured. We found that the levels of pro-caspase 1 and ASC increased with elevating of UA concentration, and the expression of NLRP3 was same in different groups (Fig. 2c). On the condition that cells lysates were extracted by A/G immunomagnetic beaded with ASC antibody, the level of NLRP3 and pro-caspase 1 elevated with increasing of UA concentration with the same expression of ASC in different groups (Fig. 2d). Therefore, we concluded that UA could induce the expression of NLRP3 complexes and activation of NLRP3 inflammasome in HUVECs.

### NLRP3 siRNA or inhibitor of caspase 1 affected the components of NLRP3 inflammasome and the expression of downstream cytokines

**Fig. 3:**
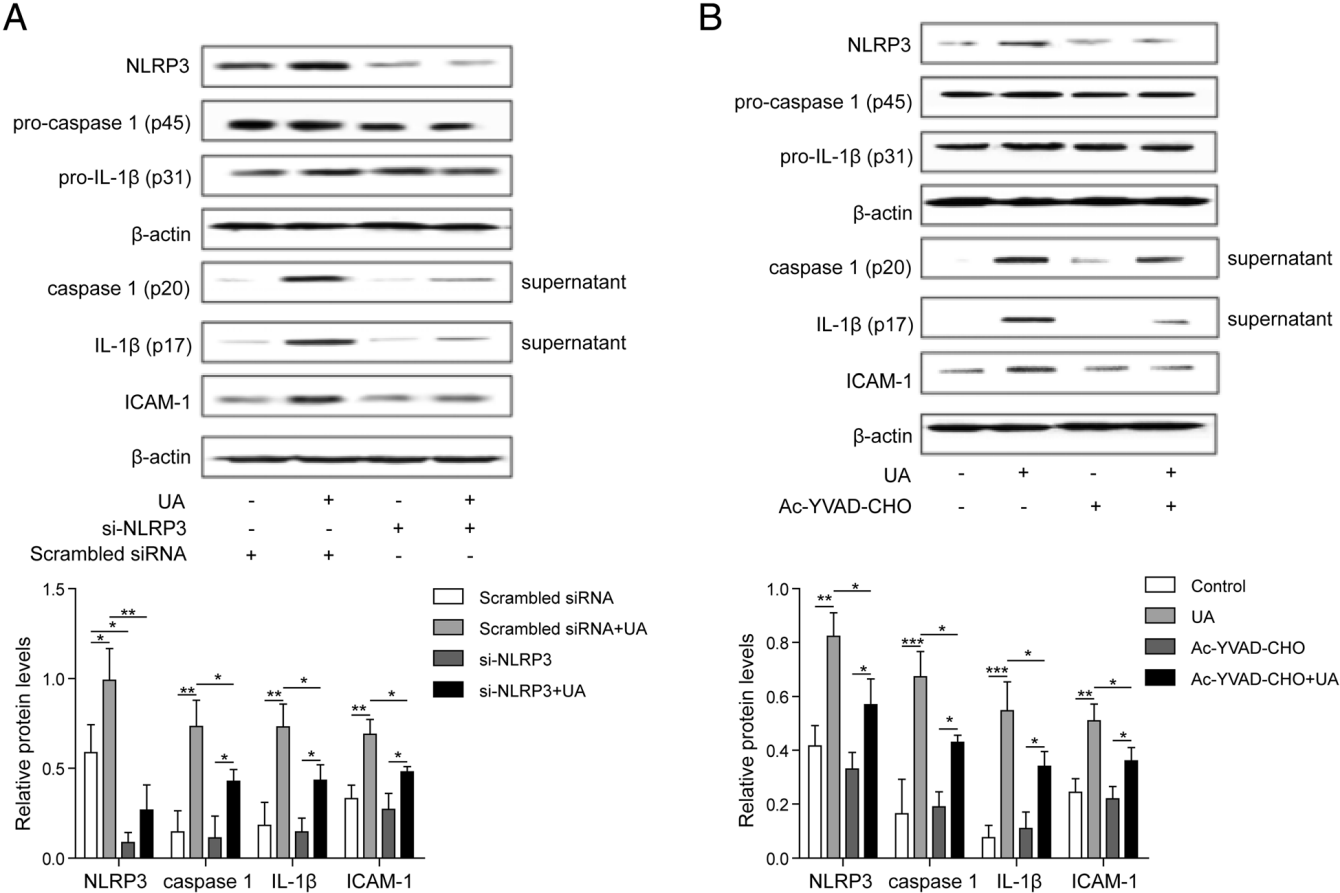
NLRP3 siRNA and inhibitor of caspase 1 affected the component of NLRP3 inflammasome and the expression of downstream cytokines. **a** After transfection of HUVECs with siRNA NLRP3, and subsequent incubation with UA (20 mg/dL) for 24 h, the expression of NLRP3, IL-1β, caspase 1, and ICAM-1 in cells or culture supernatant was measured by western blotting; **b** After 1 h pretreatment of HUVECs with caspase 1 inhibitor (10 μM Ac-YVAD-CHO), and subsequent incubation with UA (20 mg/dL) for 24 h, the expression of NLRP3, IL-1β, caspase 1, and ICAM-1 in cells or culture supernatant was measured by western blotting. *P < 0.05; **p < 0.01; ***p < 0.001

To investigate the regulation of UA on downstream cytokines expression by activating NLRP3, the NLRP3 inflammasome components and downstream cytokines expression were measured after NLRP3 siRNA or caspase 1 inhibitor treatment, respectively. We found that UA could increase the expression of caspase 1, NLRP3, IL-1β, and ICAM-1 in HUVECs, but the trends were significantly suppressed by siRNA NLRP3 treatment. However, no remarkable changes were observed in the expression of pro-IL-1β and pro-caspase 1 after siRNA NLRP3 treatment (Fig. 3a). Caspase 1 activated by the combination of NLRP3 complexes is an important regulator of the cleavage of pro-IL-1β into mature IL-1β. After the treatment of caspase 1 inhibitor (Ac-YVAD-CHO), the expression of caspase 1, IL-1β, and ICAM-1 markedly declined, but no significant difference was found in the expression of pro-IL-1β and pro-caspase 1 (Fig. 3b). Therefore, UA might regulate the level of downstream cytokines by activating NLRP3.

### UA regulated the activation of NLRP3 inflammasome by activation of ROS and K^+^ efflux

**Fig. 4:**
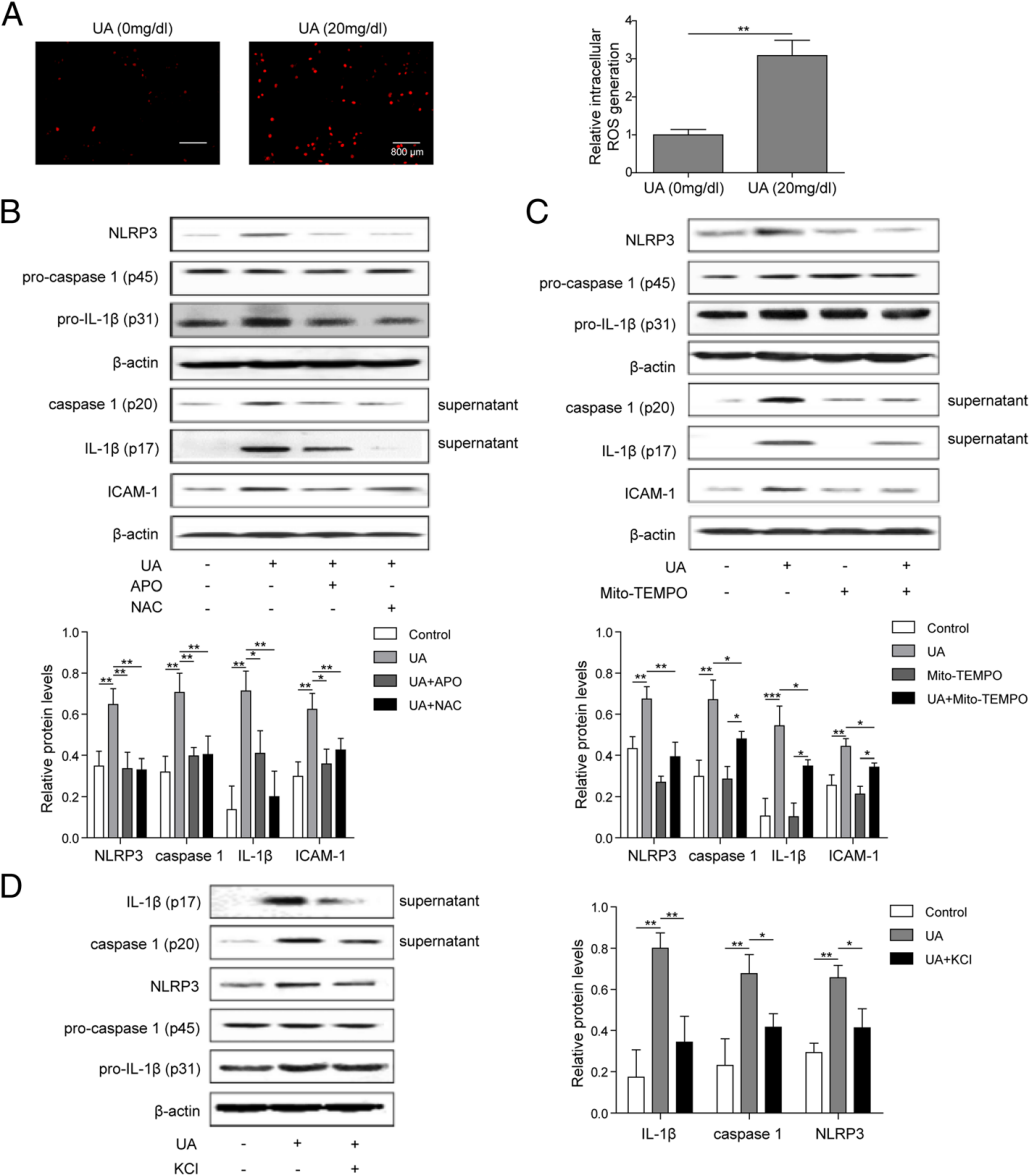
UA regulated the activation of NLRP3 inflammasome by activation of ROS and K^+^ efflux. **a** After treatment of HUVECs with UA (20 mg/dL) or normal saline for 24 h, the level of ROS in cells was measured using CM-H2DCFDA assay; **b** After pretreatment of HUVECs with APO (200 μM) or NAC (5 mM) for 1 h, and subsequent incubation with UA (20 mg/dL) for 24 h, the expression of NLRP3 and its downstream factors in cells or culture supernatant was measured by western blotting; **c** After 1 h treatment with Mito-TEMPO (500 μM), and subsequent incubation with UA (20 mg/dL) for 24 h, the expression of NLRP3 and its downstream factors in cells or culture supernatant was measured by western blotting; **d** HUVECs were treated with potassium chloride (KCl, 130 mM) for 30 min in extracellular, the medium containing K^+^ was removed. After subsequent incubation with UA (20 mg/dL) for 24 h, the expression of NLRP3 and its downstream factors in cells or culture supernatant was measured by western blotting. *P < 0.05; **p < 0.01; ***p < 0.001

To unfold whether UA regulate the activation of NLRP3 inflammasome via ROS activation or K^+^ efflux, we blocked K^+^ efflux using extracellular high concentration K^+^, and treated cells with ROS inhibitor (Apocynin, APO), ROS scavenger (N-acetylcysteine, NAC), or mitochondrial ROS inhibitor (Mito-TEMPO). Then the expression of NLRP3 and its downstream factors, caspase 1, IL-1β and ICAM-1, was measured. As shown, the ROS level in cells was significantly promoted by UA after 24 h incubation (Fig. 4a). Then we found that UA could induce the activation of caspase 1 and NLRP3, and increase the expression of IL-1β and ICAM-1, but pretreatment with APO or NAC significantly inhibited these trends (Fig. 4b). Mito-TEMPO pretreatment or high concentration of K^+^ in extracellular significantly inhibited the formation of NLRP3 inflammasome, activation of caspase 1, release of IL-1β and expression of ICAM-1 induced by UA (Fig. 4c, d). These findings suggested that UA could regulate activation of NLRP3 inflammasome and expression of inflammatory factors by activating ROS or regulating K^+^ efflux.

### UA induced the vascular endothelial injury of early stage CKD by activating NLRP3/ IL-1β pathway

To investigate the molecular mechanism of UA-induced vascular endothelial injury in early CKD, the results in cells level were further validated through animal experiments. Rats were separated randomly into four groups: Group A served as the control; Group B, in which animals only received right nephrectomy; Group C, in which animals received right nephrectomy and then gavaged with OXO (800 mg/kg, twice a day); Group D, in which animals received right nephrectomy and then gavaged with OXO (800 mg/kg, twice a day) and allopurinol (25 mg/kg, once a day).

**Fig. 5:**
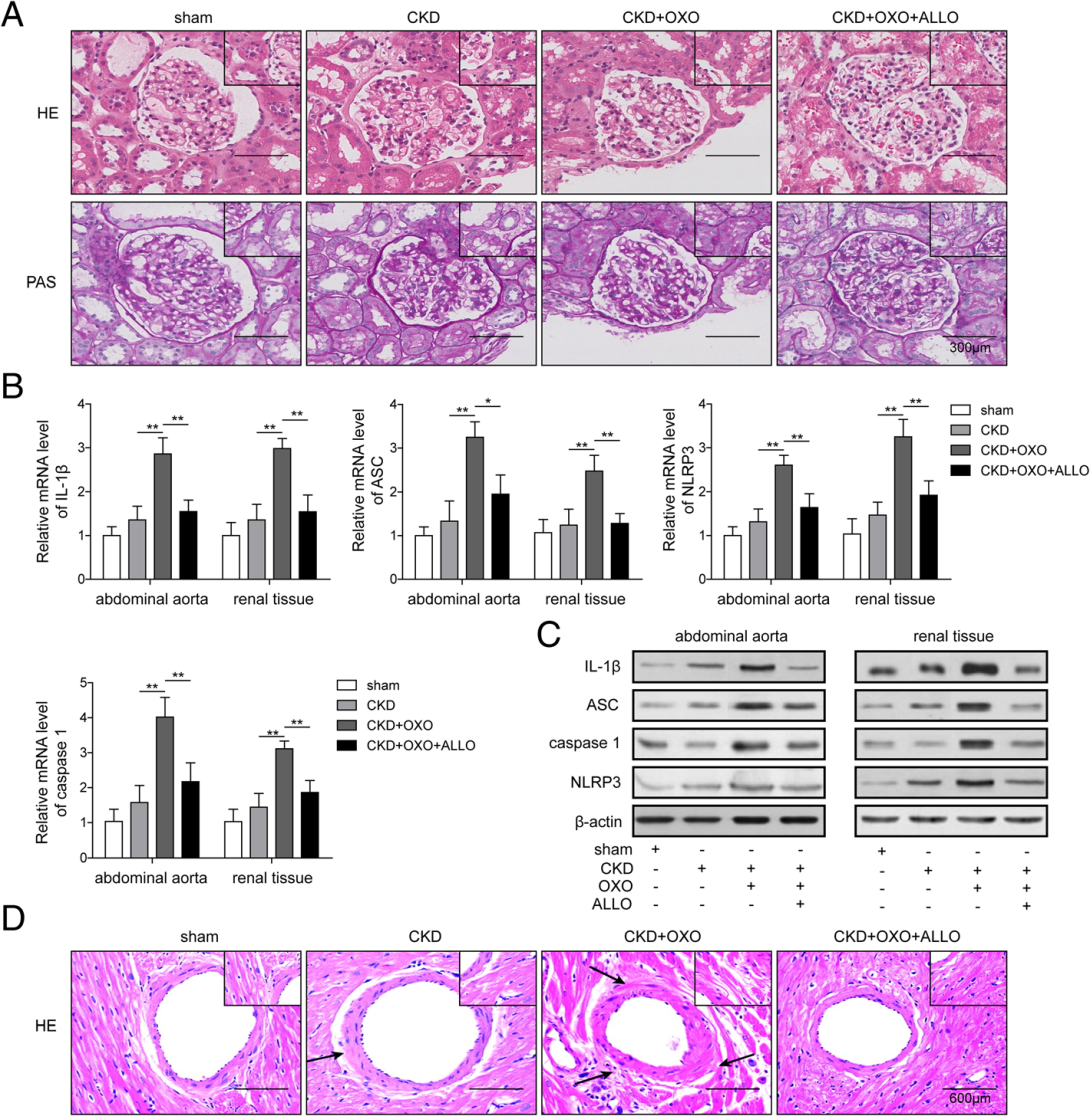
UA induced the vascular endothelial injury of early stage CKD by activating NLRP3/IL-1β pathway. **a** Glomeruli and renal tubule tissues of rats were stained by HE or PAS; **b** The mRNA expression of ASC, caspase 1, NLRP3, and IL-1β in abdominal aorta and renal tissue was measured by qPCR; **c** The protein expression of ASC, caspase 1, NLRP3, and IL-1β in abdominal aorta and renal tissue was measured by western blotting; **d** The vascular injury was investigated through HE staining. *P < 0.05; **p < 0.01; ***p < 0.001.

During the experiment, no dead rat, and no glomerular, tubule, and interstitial lesions in all groups (Fig. 5a). The serum creatinine in four groups were also no changes, but the content of serum UA in group C was significant higher than group A and group B, and allopurinol presented inhibition function on the increase of UA (Table 2). In the group C accompanied with increase of serum UA, the expressions of IL-1β, TNF-α, and ICAM-1 in rat serum were significantly higher than group A, group B, and group D (Table 3). The mRNA and protein levels of NLRP3, caspase 1, ASC, and IL-1β in group C were remarkably higher than group A and group B, and significantly decreased in group D (Fig. 5b, c). For the histopathology in the group A, we found that the structure of aorta was clear, the endothelial cells arranged closely, and there was no inflammatory cells aggregation in the vessel wall. Meanwhile, slight infiltration of inflammatory cells could be seen on the vessel wall in group B. However, edematous endothelial cells appeared foam like change, a little inflammatory cells aggregation in the wall, smooth muscle cells proliferation and structural disorder were also found in group C. Compared with group C, the lesions in group D were lighter. The proliferation of smooth muscle cells was not obvious, and the structure of cells arranged orderly in group D (Fig. 5d). We investigated the histopathology changes of five mice for each group. The histopathology changes of mice vascular were similar in the same group, and representative pictures were shown in the study. These results demonstrated that UA might induce early vascular endothelial injury by activating NLRP3/IL-1β signaling pathway.

**Table 2:** The concentration of serum creatinine and UA after different kinds of operations

Group	n	Creatinine (μM)	UA (μM)
Sham	5	30.4 ± 7.2	54.8 ± 9.4
CKD	5	31.9 ± 4.8	55.9 ± 6.1
CKD + OXO	5	31.2 ± 5.7	178.5 ± 8.4*
CKD + OXO + ALLO	5	30.7 ± 7.7	74.9 ± 6.1^#^

*, compared with group A or group B, *p* < 0.05; ^#^, compared with group C, *p* < 0.05

**Table 3:** The expression of IL-1β, TNF-α, and ICAM-1 after different kinds of operations

Cytokines	Sham	CKD	CKD + OXO	CKD + OXO + ALLO
IL-1β (pg/mL)	42.38 ± 7.96	45.72 ± 8.13	88.73 ± 5.64*	47.25 ± 6.14^#^
TNF-a (ng/mL)	163.70 ± 10.83	174.20 ± 11.24	293.10 ± 12.31*	183.60 ± 14.28^#^
ICAM-1 (ng/mL)	284.10 ± 22.64	311.50 ± 27.83	592.18 ± 98.20*	328.70 ± 76.12^#^

*, compared with group A or proupB, *p* < 0.05; ^#^, compared with group C, *p* < 0.05

## Discussion

UA is produced during the metabolism of nucleotides and adenosine triphosphate (ATP), it is the end product of purine metabolism in humans which excreted mainly by kidney [18, 19]. While UA may be an important risk factor for CKD, high serum UA content is closely associated with several adverse pathological and cellular processes, such as inflammation, endothelial dysfunction, oxidative stress, reduction of NO production and decrease of biological activity, which increases the harm of CKD [18]. In this study, we found that UA could significantly increase the level of some inflammation factors including IL-1β and ICAM-1 in HUVECs and serum of early CKD rats. The occurrence of many human diseases are related to the abnormal expression of IL-1β, which is a promising target for clinical intervention of inflammation-related diseases. ICAM-1, an important adhesion molecule and high expression in vascular endothelial cells, can promote adhesion between leukocytes and endothelial cells. The increase of IL-1β and ICAM-1 induced by UA probably account for vascular endothelium damage. Furthermore, we also observed the inflammatory cells aggregation in the wall, smooth muscle cells proliferation and structural disorder in histological level for the rats treated by nephrectomy and OXO, which could cause hyperuricemia. These findings above in vivo and in vitro identified the adverse effect of UA on vascular endothelium.

It was reported that endothelial cells damage induced by inflammatory factors plays a key role in the pathogenesis of vascular diseases [20, 21]. NLRP3 inflammasome, a multiprotein complex, can activate caspase 1 and lead to the secretion of IL-1β. The activation of NLRP3 inflammasome may lead to the development of endothelial dysfunction [22, 23] and further accelerate the injury of vascular. In this study, we have found that UA activated NLRP3 inflammasome and increased the expression of IL-1β, caspase 1, and ICAM-1 in HUVECs. In vivo, edematous endothelial cells appeared foam like change after OXO treatment, which could cause hyperuricemia. Therefore, UA may cause vascular endothelial injury by activating NLRP3 inflammasome and promoting the level of inflammatory factors.

Some reports indicated that cytoplasmic K^+^ concentration significantly influences the activation of NLRP3 inflammasome [24, 25]. The cytoplasmic K^+^ concentration of healthy cells is ~ 140–150 mM, which does not induce NLRP3 activation [26, 27]. ATP, a strong activator of NLRP3 inflammasome, could decrease the cytoplasmic K^+^ concentration by 50% [27]. Therefore, we used extracellular high concentration K^+^ for blocking K^+^ efflux, and observed the inhibition of NLRP3 inflammasome activation. Interestingly, the activation of apoptosome also were correlated with the K^+^ concentration [26]. ROS production leads to activation of NLRP3 inflammasome through release of the ROS-sensitive NLRP3 ligand thioredoxin-interacting protein (TXNIP) [27]. The interplay between ROS production and K^+^ efflux in the activation of NLRP3 inflammasome remains unclear, but low intracellular K^+^ concentration induces ROS production and vice versa [28–30]. Therefore, UA might activate NLRP3 inflammasome by activating ROS or promoting K^+^ efflux.

## Conclusions

In the present study, UA could activate NLRP3 inflammasome and increase the expression of some inflammatory factors including ICAM-1 and IL-1β in both in vitro and in vivo early CKD model. We further indicated that UA activated NLRP3 inflammasome by activating ROS or regulating K^+^ efflux. Therefore, UA may induce the vascular endothelial injury by activating NLRP3/IL-1β pathway. These findings provide a new insight into the adverse effect of UA on vascular endothelial cells, and new promising therapeutic targets aiming at early-stage CKD may be developed by regulating NLRP3/IL-1β pathway.

## Data Availability

The datasets used and/or analyzed during this study are available from the corresponding author on reasonable request.

## Abbreviations

APO: Apocynin
ASC: Apoptosis­associated speck­like protein
ATP: Adenosine triphosphate
CKD: Chronic kidney disease
HE: Hematoxylin-eosin
HUVECs: Human umbilical vein endothelial cells
ICAM-1: Intercellular adhesion molecule-1
IL-1β: Interleukin-1 beta
IL-6: Interleukin-6
Mito-TEMPO: Mitochondrial ROS inhibitor
NAC: N-acetylcysteine
NLRP3: NLR family pyrin domain-containing 3
OXO: Potassium oxonate
PAS: Periodic acid-Schiff
qPCR: Quantitative real-time PCR
TNF-α: Tumor necrosis factor-alpha
TXNIP: Thioredoxin-interacting protein
UA: Uric acid
VCAM-1: Vascular adhesion molecule-1

## References

[1] Qiu C, Huang S, Park J, Park Y, Ko YA, Seasock MJ (2018). Renal compartment-specific genetic variation analyses identify new pathways in chronic kidney disease. Nat Med.

[2] Ko S, Venkatesan S, Nand K, Levidiotis V, Nelson C, Janus E (2018). International statistical classification of diseases and related health problems coding underestimates the incidence and prevalence of acute kidney injury and chronic kidney disease in general medical patients. Intern Med J.

[3] Goicoechea M, de Vinuesa SG, Verdalles U, Ruiz-Caro C, Ampuero J, Rincon A (2010). Effect of allopurinol in chronic kidney disease progression and cardiovascular risk. Clin J Am Soc Nephrol.

[4] Toyama T, Furuichi K, Shimizu M, Hara A, Iwata Y, Sakai N (2015). Relationship between serum uric acid levels and chronic kidney disease in a Japanese cohort with Normal or mildly reduced kidney function. PLoS One.

[5] Liu WC, Hung CC, Chen SC, Yeh SM, Lin MY, Chiu YW (2012). Association of hyperuricemia with renal outcomes, cardiovascular disease, and mortality. Clin J Am Soc Nephrol.

[6] Madero M, Sarnak MJ, Wang X, Greene T, Beck GJ, Kusek JW (2009). Uric acid and long-term outcomes in CKD. Am J Kidney Dis.

[7] Suliman ME, Johnson RJ, Garcia-Lopez E, Qureshi AR, Molinaei H, Carrero JJ (2006). J-shaped mortality relationship for uric acid in CKD. Am J Kidney Dis.

[8] Go AS, Chertow GM, Fan D, McCulloch CE, Hsu CY (2004). Chronic kidney disease and the risks of death, cardiovascular events, and hospitalization. N Engl J Med.

[9] Kanbay M, Afsar B, Siriopol D, Unal HU, Karaman M, Saglam M (2016). Relevance of uric acid and asymmetric dimethylarginine for modeling cardiovascular risk prediction in chronic kidney disease patients. Int Urol Nephrol.

[10] Kuo KL, Hung SC, Lee TS, Tarng DC (2014). Iron sucrose accelerates early atherogenesis by increasing superoxide production and upregulating adhesion molecules in CKD. J Am Soc Nephrol.

[11] Liang WY, Zhu XY, Zhang JW, Feng XR, Wang YC, Liu ML (2015). Uric acid promotes chemokine and adhesion molecule production in vascular endothelium via nuclear factor-kappa B signaling. Nutr Metab Cardiovasc Dis.

[12] Sanchez-Lozada LG, Lanaspa MA, Cristobal-Garcia M, Garcia-Arroyo F, Soto V, Cruz-Robles D (2012). Uric acid-induced endothelial dysfunction is associated with mitochondrial alterations and decreased intracellular ATP concentrations. Nephron Exp Nephrol.

[13] Zhou T, Xiang DK, Li SN, Yang LH, Gao LF, Feng C (2018). MicroRNA-495 ameliorates cardiac microvascular endothelial cell injury and inflammatory reaction by suppressing the NLRP3 Inflammasome signaling pathway. Cell Physiol Biochem.

[14] Wan X, Xu C, Lin Y, Lu C, Li D, Sang J (2016). Uric acid regulates hepatic steatosis and insulin resistance through the NLRP3 inflammasome-dependent mechanism. J Hepatol.

[15] Gross CJ, Mishra R, Schneider KS, Medard G, Wettmarshausen J, Dittlein DC (2016). K(+) efflux-independent NLRP3 Inflammasome activation by small molecules targeting mitochondria. Immunity..

[16] Meng X, Fei D, Liu M, Yang S, Song N, Jiang L (2017). Carbon monoxide-releasing molecule-2 suppresses thrombomodulin and endothelial protein C receptor expression of human umbilical vein endothelial cells induced by lipopolysaccharide in vitro. Medicine (Baltimore).

[17] Bao X, Wang Y, Wei C, Zhang Q (2014). Effects of uric acid on hearts of rats with chronic kidney disease. Am J Nephrol.

[18] Kanbay M, Segal M, Afsar B, Kang DH, Rodriguez-Iturbe B, Johnson RJ (2013). The role of uric acid in the pathogenesis of human cardiovascular disease. Heart..

[19] Kobayashi T, Nakagome K, Noguchi T, Kobayashi K, Ueda Y, Soma T (2017). Elevated uric acid and adenosine triphosphate concentrations in bronchoalveolar lavage fluid of eosinophilic pneumonia. Allergol Int.

[20] Yuan H, Ma J, Li T, Han X (2018). MiR-29b aggravates lipopolysaccharide-induced endothelial cells inflammatory damage by regulation of NF-kappaB and JNK signaling pathways. Biomed Pharmacother.

[21] Ruan W, Xu JM, Li SB, Yuan LQ, Dai RP (2012). Effects of down-regulation of microRNA-23a on TNF-alpha-induced endothelial cell apoptosis through caspase-dependent pathways. Cardiovasc Res.

[22] Kim JK, Jin HS, Suh HW, Jo EK (2017). Negative regulators and their mechanisms in NLRP3 inflammasome activation and signaling. Immunol Cell Biol.

[23] Boini KM, Hussain T, Li PL, Koka S (2017). Trimethylamine-N-oxide instigates NLRP3 Inflammasome activation and endothelial dysfunction. Cell Physiol Biochem.

[24] Lee GS, Subramanian N, Kim AI, Aksentijevich I, Goldbach-Mansky R, Sacks DB (2012). The calcium-sensing receptor regulates the NLRP3 inflammasome through Ca2+ and cAMP. Nature..

[25] Zhang Y, Rong H, Zhang FX, Wu K, Mu L, Meng J (2018). A membrane potential- and Calpain-dependent reversal of Caspase-1 inhibition regulates canonical NLRP3 Inflammasome. Cell Rep.

[26] Karki P, Seong C, Kim JE, Hur K, Shin SY, Lee JS (2007). Intracellular K(+) inhibits apoptosis by suppressing the Apaf-1 apoptosome formation and subsequent downstream pathways but not cytochrome c release. Cell Death Differ.

[27] Tschopp J, Schroder K (2010). NLRP3 inflammasome activation: the convergence of multiple signalling pathways on ROS production?. Nat Rev Immunol.

[28] Kim MJ, Ciani S, Schachtman DP (2010). A peroxidase contributes to ROS production during Arabidopsis root response to potassium deficiency. Mol Plant.

[29] He L, Dinger B, Sanders K, Hoidal J, Obeso A, Stensaas L (2005). Effect of p47phox gene deletion on ROS production and oxygen sensing in mouse carotid body chemoreceptor cells. Am J Physiol Lung Cell Mol Physiol.

[30] Xia X, Lu B, Dong W, Yang B, Wang Y, Qin Q (2018). Atypical Gasdermin D and mixed lineage kinase domain-like protein leakage aggravates Tetrachlorobenzoquinone-induced nod-like receptor protein 3 Inflammasome activation. Chem Res Toxicol.

